# Identification of a *S*-(2-succino)cysteine breakdown pathway that uses a novel *S*-(2-succino) lyase

**DOI:** 10.1016/j.jbc.2022.102639

**Published:** 2022-10-27

**Authors:** Katie B. Hillmann, Madeline E. Goethel, Natalie A. Erickson, Thomas D. Niehaus

**Affiliations:** The Department of Plant and Microbial Biology, University of Minnesota, St Paul, Minnesota, USA

**Keywords:** cysteine, fumarate, succination, 2SC, *S*-(2-succino)cysteine, oncometabolite, metabolite damage, metabolite repair, 2SC, *S*-(2-succino)cysteine, *2SL*, *S*-(2-succino) lyase, 2SNAC, *N*-acetyl-2SC, Cys, cysteine, DTT, dithiothreitol, GSH, glutathione, NAC, *N*-acetylcysteine

## Abstract

Succination is the spontaneous reaction between the respiratory intermediate fumarate and cellular thiols that forms stable *S*-(2-succino)-adducts such as *S*-(2-succino)cysteine (2SC). 2SC is a biomarker for conditions associated with elevated fumarate levels, including diabetes, obesity, and certain cancers, and succination likely contributes to disease progression. *Bacillus subtilis* has a *yxe* operon-encoded breakdown pathway for 2SC that involves three distinct enzymatic conversions. The first step is *N*-acetylation of 2SC by YxeL to form *N*-acetyl-2SC (2SNAC). YxeK catalyzes the oxygenation of 2SNAC, resulting in its breakdown to oxaloacetate and *N*-acetylcysteine, which is deacetylated by YxeP to give cysteine. The monooxygenase YxeK is key to the pathway but is rare, with close homologs occurring infrequently in prokaryote and fungal genomes. The existence of additional 2SC breakdown pathways was not known prior to this study. Here, we used comparative genomics to identify a *S*-(*2*-*succino*) *lyase* (*2SL*) that replaces *yxeK* in some *yxe* gene clusters. *2SL* genes from *Enterococcus italicus* and *Dickeya dadantii* complement *B. subtilis yxeK* mutants. We also determined that recombinant 2SL enzymes efficiently break down 2SNAC into fumarate and *N*-acetylcysteine, can perform the reverse reaction, and have minor activity against 2SC and other small molecule thiols. The strong preferences both YxeK and 2SL enzymes have for 2SNAC indicate that 2SC acetylation is a conserved breakdown step. The identification of a second naturally occurring 2SC breakdown pathway underscores the importance of 2SC catabolism and defines a general strategy for 2SC breakdown involving acetylation, breakdown, and deacetylation.

The intermediary metabolite fumarate is a soft electrophile that spontaneously and readily reacts with soft nucleophiles, predominantly cellular thiols (1, 2), to produce stable *S*-(2-succino)-adducts in a reaction called succination (3, 4). Fumarate can react with the thiolate anion of cysteine to form *S*-(2-succino)cysteine (2SC) (Fig. 1*A*). However, small molecule thiols such as glutathione (GSH) or cysteine residues of proteins are more likely targets of succination under physiological conditions because of their abundance and because their sulfhydryl groups can be more acidic and reactive than that of free cysteine (3, 4, 5, 6). 2SC also denotes succinated cysteine residues occurring on proteins or GSH. Hundreds of proteins can be succinated *in vivo* (7) and at least some with functional cysteine residues, including glyceraldehyde-3-phosphate dehydrogenase (8, 9), aconitase (10), actin, and tubulin (11, 12), and chaperone proteins (12), are inactivated or functionally impaired due to the 2SC modification. The degree that 2SC-modified proteins form is directly related to cellular fumarate levels (3). Thus, 2SC is a biomarker for conditions that result in fumarate build-up and increased succination of cellular thiols, such as hyperglycemia associated with type 2 diabetes and obesity (13, 14, 15, 16). Succination considerably increases as a result of inactivation of fumarate hydratase (17, 18), a key enzyme in controlling fumarate levels. Germline mutations in fumarate hydratase predispose humans to hereditary leiomyomatosis and renal cell cancer syndrome, likely due to complications arising from widespread succination (19, 20, 21). The deleterious effects of protein (16, 22, 23, 24) and GSH (25) succination likely contributes to the pathogenesis of metabolic diseases associated with elevated fumarate (26). Succination is thus a well-defined posttranslational modification of proteins and is an example of metabolite damage of macromolecules, which is increasingly implicated in the progression of aging and neurodegeneration (27, 28, 29).

**Figure 1:**
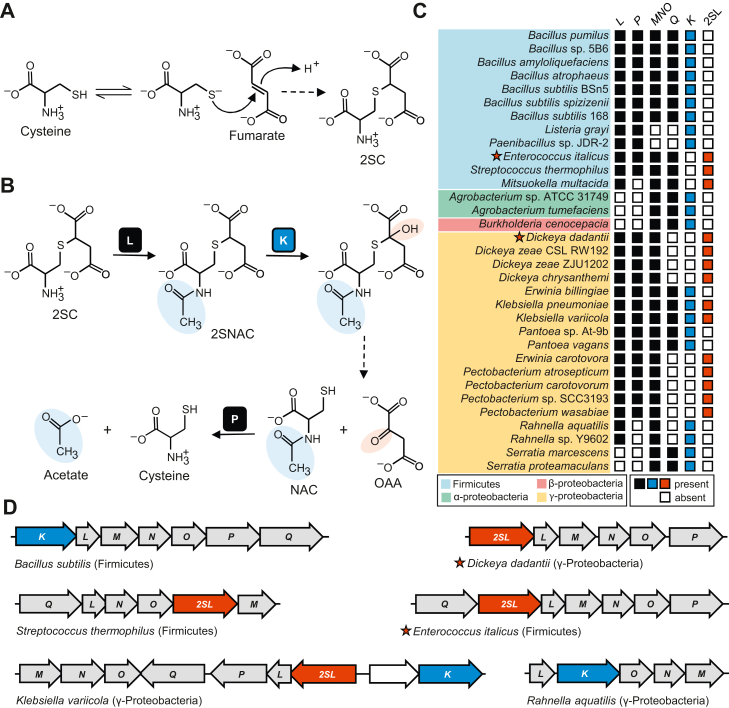
**2SC metabolism and the genomic organization and distribution of 2SC metabolic genes in prokaryotes.***A*, cellular thiols such as cysteine react with fumarate to form 2SC. *B*, 2SC breakdown pathway by Yxe enzymes (L, K, and P) in *Bacillus subtilis*. *C*, distribution of *yxe* locus genes in taxonomically diverse prokaryotes. The presence or absence of *yxe* genes that cluster on the chromosome are indicated with filled or empty boxes, respectively. *2SL* genes characterized in this study are marked with a *star*. *D*, genomic organization of *yxe* locus genes (*gray*) in representative genomes. *2SL* (*red*) sometimes replaces *yxeK* (*blue*) in the locus. 2SC, *S*-(2-succino)cysteine; *2SL*, *S*-(2-succino) lyase.

Although many studies have focused on the macromolecular targets of succination and the physiological consequences of 2SC buildup, little is known about the metabolic fate of 2SC after it is formed, particularly in eukaryotes. Prior to this study, only one 2SC breakdown pathway has been characterized in *Bacillus subtilis* (30). The pathway is encoded by the *S-(2-succino)cysteine metabolism* (*scm*) operon (formerly the *yxe* operon; we use the previous *yxe* denotation here) and consists of three enzymatic steps (Fig. 1*B*). The breakdown pathway is initiated by the acetyltransferase YxeL, which *N*-acetylates 2SC to form *N*-acetyl-2SC (2SNAC) (30). The FMN-dependent monooxygenase YxeK hydroxylates 2SNAC, causing its breakdown to oxaloacetate and *N*-acetylcysteine (NAC) (30, 31). In the final step, NAC gets deacetylated by YxeP to release free cysteine (30). The *yxe* gene cluster is found in a wide range of taxonomically diverse bacteria but is limited to a small proportion of total species (30). Furthermore, the monooxygenase YxeK, which catalyzes the key breakdown step in the pathway, occurs infrequently in prokaryote and fungal genomes.

Because of the widespread occurrence of succination and its negative effects on physiology, we predicted that strategies to deal with 2SC exist in addition to the *B. subtilis* breakdown pathway. Here, we present biochemical and genetic evidence for a second 2SC breakdown pathway that uses the same general strategy of acetylation, breakdown, and deacetylation to recover cysteine from 2SC, except that the breakdown step is mediated by a lyase instead of the monooxygenase YxeK.

## Results

### Bioinformatic identification of an alternative 2SC breakdown pathway

We used the SEED database and its tools (32) to survey the distribution of the *yxe* locus in a representative set of ∼2000 microbial genomes (33). For our analysis, we defined the *yxe* locus (or gene cluster) as a group of genes clustered on the chromosome that encode at least three of the five enzymatic components of the *B. subtilis* operon (YxeK, 2SNAC monooxygenase; YxeL, 2SC acetyltransferase; YxeMNO, three components of an ABC-transporter; YxeP, NAC deacetylase; and YxeQ, unknown function). Using this criteria, we identified 102 organisms that have the *yxe* locus (a representative subset is shown in Fig. 1*C*). Unexpectedly, only 67 of the 102 *yxe* loci encode the monooxygenase YxeK, even though it catalyzes the critical breakdown step in 2SC catabolism. The 35 *yxe* locus-containing genomes without *yxeK* had no close homolog of *yxeK* (>50% identity to the *B. subtilis* enzyme) present elsewhere in the genome (Fig. 1*C*). This implies that either the *yxe* locus has alternate functions apart from 2SC catabolism or that a functionally redundant gene replaces *yxeK* in genomes where it is lacking. Inspection of *yxe* loci revealed that in some genomes lacking *yxeK*, a gene (here name *S*-(*2-succino*) *lyase*, *2SL*) was present that encoded a protein of the lyase class I conserved protein domain family (cd01334) (34) (Fig. 1*D*). *2SL* occurred in the *yxe* loci of several taxonomically diverse bacteria and in various orientations (Fig. 1, *C* and *D*), indicating that *2SL* has a functional role related to the *yxe* locus. *2SL* was part of the *yxe* locus in 27 of the 102 *yxe* locus-containing organisms, and *2SL* and *yxeK* are essentially inversely distributed and rarely (three times) occur together (Fig. 1*C*), which is consistent with their having redundant functions. Enzymes of the lyase class I conserved protein domain family in which 2SL belongs typically catalyze a beta-elimination reaction involving cleavage of an *O*-succino or *N*-succino moiety to release fumarate as one of the products (35, 36). Although a lyase acting on a *S*-succino moiety has not been described, it is conceivable that this enzyme family could be involved in breakdown of 2SC or 2SNAC. In summary, inclusion of *2SL* in the *yxe* locus, the opposite distribution of *2SL* and *yxeK*, and the fumarate lyase activity of many members of the lyase class I protein family are all consistent with 2SL catalyzing the breakdown step of 2SC catabolism. Interestingly, 11 of the 102 *yxe* loci do not contain *yxeK* or *2SL*, suggesting additional genes may be involved in 2SC breakdown.

### 2SL enzymes allow a *B. subtilis ΔyxeK* mutant to use 2SC as a sulfur source

**Figure 2:**
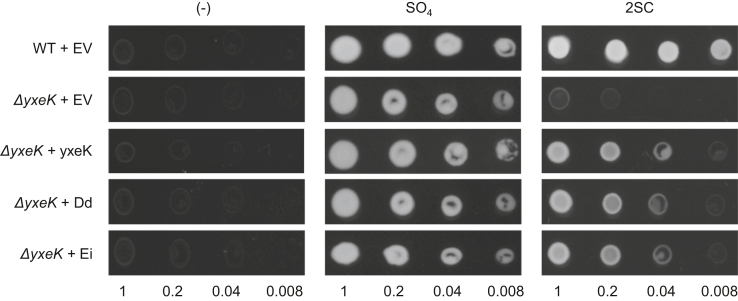
**Expression of *2SL* genes enables a *B. subtilis ΔyxeK* mutant to grow on 2SC.***B. subtilis* 168 harboring an empty pHCMC04 vector (EV) and *B. subtilis ΔyxeK* mutants harboring an empty pHCMC04 vector or pHCMC04 containing *B. subtilis yxeK*, *D. dadantii* (Dd) *2SL*, or *E. italicus* (Ei) *2SL* coding sequences were grown overnight at 37 °C in 5 ml LB medium with 5 μg ml^−1^ chloramphenicol. Cells were washed twice with ED minimal medium lacking sulfur, diluted to absorbances (600 nm) of 1.0, 0.2, 0.04, and 0.008, and 5 μl and was spotted on ED minimal medium plates containing 0.2% xylose and either no added sulfur (−) or 2 mM sulfate (SO_4_) or 0.25 mM 2SC as the sole sulfur source. Plates were incubated for 16 h at 37 °C. 2SC, *S*-(2-succino)cysteine; *2SL*, *S*-(2-succino) lyase.

To determine whether 2SL is involved in 2SC metabolism, we tested *2SL* genes from two prokaryotes for the ability to complement *B. subtilis ΔyxeK* mutants, which lack their native gene responsible for 2SNAC breakdown and are not able to use 2SC as a sulfur source (30). We selected genes from *Enterococcus italicus* of the phylum Firmicutes and *Dickeya dadantii* of the phylum Proteobacteria because they belong to taxonomically diverse bacteria and because their *yxe* loci are similar to the previously characterized *B. subtilis yxe* operon except that *yxeK* has been replaced with *2SL* (Fig. 1, *C* and *D*). The *2SL* coding sequences were cloned into the replicating pHCMC04 plasmid, which has been used previously in functional complementation studies in *B. subtilis* (37). Wildtype *B. subtilis* 168 harboring an empty pHCMC04 plasmid and a *B. subtilis ΔyxeK* mutant harboring either empty plasmid or pHCMC04 containing *D. dadantii 2SL*, *E. italicus 2SL*, or *B. subtilis yxeK* were plated on ED minimal medium lacking sulfur without or with 2SC or sulfate supplementation. No strain grew on medium without added sulfur (Fig. 2), confirming that our base ED medium lacked sufficient sulfur to sustain growth. When sulfate was added to reconstitute standard ED minimal medium, all five strains grew similarly (Fig. 2). As expected, wildtype *B. subtilis* harboring an empty plasmid grew well on 2SC, but the *ΔyxeK* mutant harboring an empty plasmid showed virtually no sign of growth (Fig. 2). When either *E. italicus* or *D. dadantii 2SL* was expressed in the *ΔyxeK* mutant, growth on 2SC was readily observed and was similar to the growth observed when *yxeK* was expressed in the *ΔyxeK* mutant (Fig. 2). These results show that *2SL* genes can functionally complement *yxeK* in *B. subtilis* and indicate that 2SL is involved in 2SC breakdown.

### 2SL releases fumarate from 2SNAC and 2SC

**Figure 3:**
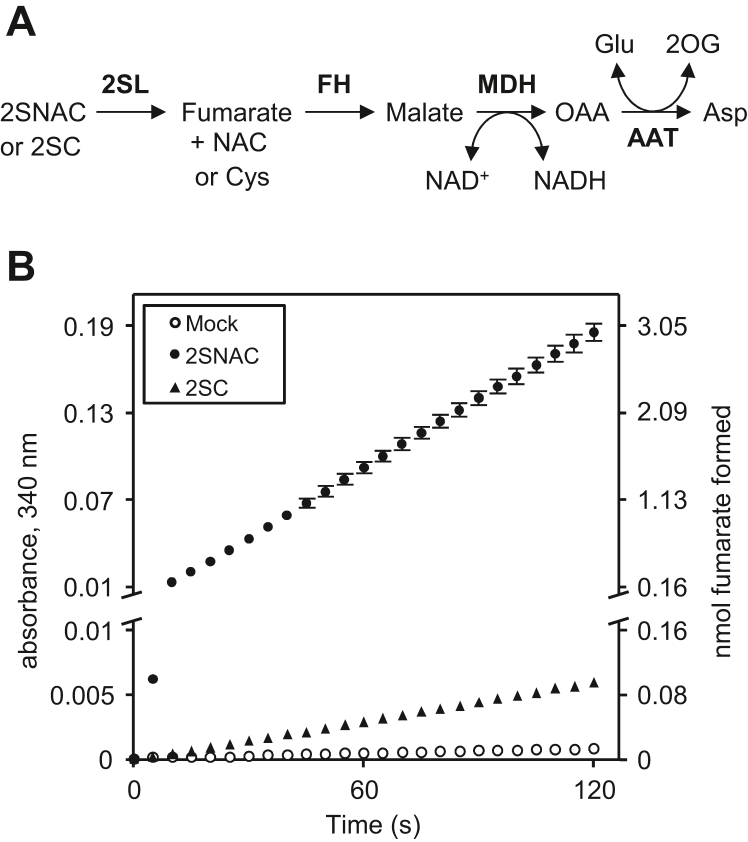
**Spectrophotometric detection of 2SL enzyme activity using 2SNAC and 2SC as substrates.***A*, schematic representation of the reporter assay. Enzymes are indicated with *bold font*. AAT, aspartate transaminase; 2OG, 2-oxoglutarate; FH, fumarate hydratase; MDH, malate dehydrogenase; OAA, oxaloacetate. *B*, assays (100 μl total) contained 100 mM Tris-HCl, pH 8.0, 40 mM Glu, 2 mM NAD^+^, 6 U MDH, 1.1 U FH, 2.5 U AAT, 1 μg *E italicus* 2SL and were started by adding either 14 mM 2SC, 1.8 mM 2SNAC, or H_2_O (mock). Data represent mean and S.E.M. of three independent experiments. S.E.M. smaller than data points are not shown. The y-axis scale changes at the break. 2SC, *S*-(2-succino)cysteine; 2SL, *S*-(2-succino) lyase; 2SNAC, *N*-acetyl-2SC.

The results of our functional complementation assays (Fig. 2) indicated that 2SL enzymes are involved in 2SC breakdown but did not provide much insight into the reaction mechanism. Based on the beta-elimination reactions catalyzed by many members of the lyase class I protein family, we predicted that 2SL enzymes catalyze cleavage of the *S*-(2-succino) moiety of 2SC (or 2SNAC) causing the release of fumarate. To test this, we used a well-defined reporter assay (Fig. 3*A*) to measure fumarate (38). The reporter assay uses three commercially available enzymes and is based on the stoichiometric reduction of NAD^+^ to NADH, which can be measured by its distinctive absorbance at 340 nm. First, fumarate hydratase converts fumarate to malate. Malate dehydrogenase then couples the oxidation of malate to oxaloacetate with the reduction of NAD^+^ to NADH. Since malate dehydrogenase is reversible, aspartate aminotransferase is included to convert oxaloacetate to aspartate, thus preventing the back reaction of malate dehydrogenase from occurring.

*2SL* coding sequences from *D. dadantii* and *E. italicus* were cloned into pET28b to facilitate expression of enzymes with *N*-terminal hexahistidine-tags, which are commonly used to purify active lyase class I proteins (39, 40). Recombinant enzymes were produced in *Escherichia coli* and purified to near homogeneity using immobilized nickel affinity chromatography (Fig. S1). When 1 μg of *E. italicus* 2SL enzyme was added to the reporter assay described above with no added substrate (2SC or 2SNAC), the absorbance at 340 nm was practically stable over the course of a few minutes (Fig. 3*B*). Addition of 2SC caused the absorbance at 340 nm to steadily increase (Fig. 3*B*). When 2SNAC was assayed, the increase in absorbance at 340 nm was ∼40-fold higher than that with 2SC (Fig. 3*B*). Similar trends were observed with *D. dadantii* 2SL. These results indicate that 2SL enzymes promote the release of fumarate from 2SC and 2SNAC, with 2SNAC being the far better substrate. Control assays confirmed that 2SNAC lyase activity is catalyzed by 2SL enzymes and that this activity is absent in *E. coli* lysates unless recombinant 2SL enzymes are expressed (Fig. S2).

### 2SL catalyzes reversible breakdown of 2SC and 2SNAC

**Figure 4:**
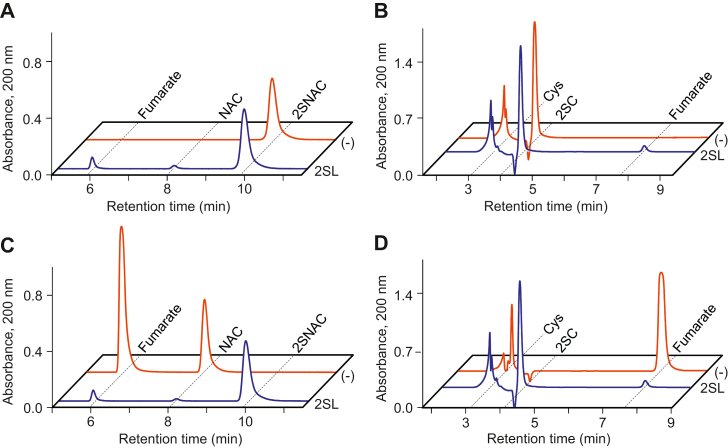
**2SL enzymes catalyze the reversible cleavage of 2SNAC and 2SC.** Assays (100 μl total) contained 50 mM Tris-HCl, pH 7.5, and either 4 mM 2SNAC (*A*), 4 mM 2SC (*B*), 4 mM fumarate and 4 mM NAC (C), or 4 mM fumarate and 4 mM cysteine (*D*) and were started by adding 1.0 μg *E italicus* 2SL (*blue trace*) or an equivalent volume of enzyme storage buffer as a control (*red trace*). After incubation for either 15 (2SNAC) or 60 (2SC) minutes at 37 °C, reactions were stopped by adding 5 μl of 1 M HCl and analyzed by HPLC. 2SC, 2SNAC, cysteine, fumarate, and NAC retention times are marked by *dashed lines*. Using *D. dadantii* 2SL gave nearly identical results. 2SC, *S*-(2-succino)cysteine; *2SL*, *S*-(2-succino) lyase; 2SNAC, *N*-acetyl-2SC; NAC, *N*-acetylcysteine.

Since we detected fumarate formation when 2SL enzymes were incubated with 2SC or 2SNAC, we assumed that the substrates were cleaved to fumarate and either cysteine or NAC, respectively. To thoroughly investigate the reactions catalyzed by 2SL enzymes, we monitored enzyme assays with high performance liquid chromatography (HPLC) under conditions allowing the separation and quantification of all substrates and products. When *E. italicus* 2SL was incubated with 2SNAC, fumarate and NAC were formed in equal amounts and 2SNAC levels correspondingly decreased (Fig. 4*A*). When 2SC was assayed, fumarate and cysteine were formed and 2SC correspondingly decreased (Fig. 4*B*). Although we were able to detect the expected reaction products, they were formed in small amounts, and increasing either the amount of 2SL added to the assay or the reaction time did not increase the levels of products formed. This indicated that the reactions were reversible and had reached equilibrium. To test the reverse reaction, 2SL was incubated with equimolar amounts of fumarate and NAC, which caused these compounds to decrease substantially and a stoichiometric amount of 2SNAC to be formed (Fig. 4*C*). Similarly, incubation of 2SL with cysteine and fumarate caused a near depletion of both substrates and a large accumulation of 2SC (Fig. 4*D*). The same substrate(s): products(s) ratio was observed in the breakdown and synthesis reactions of 2SNAC (Fig. 4, *A* and *C*) and 2SC (Fig. 4, *B* and *D*), further confirming that the reaction catalyzed by 2SL is reversible. The equilibrium constant *K*_*c*_ (2SNAC → NAC + fumarate) was determined to be 0.0046 ± 0.0002 for *D. dadantii* 2SL and 0.0067 ± 0.0001 for *E. italicus* 2SL.

### Kinetic analysis of 2SL enzymes

We determined the kinetic parameters of the forward reactions (*i.e.*, breakdown reactions; 2SNAC or 2SC → fumarate + NAC or cysteine) catalyzed by 2SL enzymes so we could better assess whether these activities are physiologically relevant. The spectrophotometric-based reporter assay described above was used except that conditions were optimized to ensure reliable values were obtained (see Experimental procedures). Both *E. italicus* and *D. dadantii* 2SL enzymes had similar kinetic parameters regarding 2SC breakdown, having a *K*_*m*_ for 2SC in the ∼10 mM range and similarly low turnover numbers (Table 1). With respect to 2SNAC breakdown, both enzymes had a similar *K*_*m*_ for 2SNAC, which was ∼10-fold lower than their *K*_*m*_ for 2SC (Table 1). However, the *E. italicus* enzyme had ∼6-fold higher turnover number and was >5-fold more catalytically efficient than the *D. dadantii* enzyme (Table 1). The catalytic efficiencies of 2SNAC breakdown were ∼60-fold and ∼320-fold higher than that of 2SC breakdown for the *D. dadantii* and *E. italicus* enzymes, respectively (Table 1). These results confirm that the substrate 2SNAC is strongly preferred over 2SC and show that *E. italicus* 2SL breaks down 2SNAC at a higher velocity than the *D. dadantii* enzyme.

**Table 1:** Kinetic parameters for recombinant 2SL proteins

Enzyme	Reaction	Substrate	Kinetic parameter			
			*K*_*m*_ (mM)	*V*_*max*_ (μmol min^−1^ mg^−1^)	*K*_*cat*_ (s^−1^)	*K*_*cat*_*/K*_*m*_ (mM^−1^s^−1^)
Dd	2SNAC → NAC + Fumarate	2SNAC	1.20 ± 0.22	0.40 ± 0.06	0.35 ± 0.05	0.29
	NAC + Fumarate → 2SNAC	NAC	0.69 ± 0.05	27.6 ± 4.2	24.2 ± 3.7	35
		Fumarate	3.24 ± 0.30			7.5
	2SC → Cysteine + Fumarate	2SC	12.3 ± 1.1	0.069 ± 0.005	0.060 ± 0.004	0.0049
	Cysteine + Fumarate → 2SC	Cysteine	19.0 ± 1.9	11.1 ± 0.5	9.7 ± 0.4	0.51
Ei	2SNAC → NAC + Fumarate	2SNAC	1.40 ± 0.10	2.42 ± 0.24	2.12 ± 0.21	1.51
	NAC + Fumarate → 2SNAC	NAC	0.39 ± 0.03	105.0 ± 8.1	92.1 ± 7.1	236
		Fumarate	0.23 ± 0.02			394
	2SC → Cysteine + Fumarate	2SC	10.7 ± 2.3	0.058 ± 0.005	0.051 ± 0.004	0.0047
	Cysteine + Fumarate → 2SC	Cysteine	17.8 ± 3.6	11.9 ± 1.7	10.4 ± 1.5	0.58

Data represents mean and S.E.M of at least three independent replicates using fresh preparations of recombinant *D. dadantii* (Dd) and *E. italicus* (Ei) 2SL enzyme.

Although the breakdown reaction is expected to occur physiologically, the reverse reaction occurs readily *in vitro* (Fig. 4). We determined the kinetic parameters of the reverse reactions (*i.e.*, synthesis reactions; fumarate + NAC or cysteine → 2SNAC or 2SC) catalyzed by 2SL so that we could compare the kinetics of breakdown and synthesis reactions. Optimized assays (see Experimental procedures) in the reverse direction were stopped by adjusting the pH to ∼2.5 with HCl followed by HPLC analysis. With respect to 2SNAC synthesis, the turnover number for the *E. italicus* enzyme was ∼4-fold higher than that of the *D. dadantii* enzyme (Table 1). Both enzymes had a similar *K*_*m*_ for NAC, but the *D. dadantii* enzyme had ∼14-fold higher *K*_*m*_ for fumarate (Table 1). Largely due to its low affinity for fumarate, the *D. dadantii* enzyme was ∼53-fold less catalytically efficient at synthesizing 2SNAC than *E. italicus* 2SL, with respect to fumarate (Table 1). Both *E. italicus* and *D. dadantii* 2SL enzymes had similar kinetic parameters regarding 2SC synthesis, having a *K*_*m*_ for cysteine in the ∼20 mM range and low turnover numbers (Table 1). These results show that acetylated reactants are strongly preferred in both breakdown and synthesis reactions catalyzed by 2SL enzymes. Although the *D*. *dadantii* enzyme is less catalytically efficient at catalyzing the physiologically relevant 2SNAC breakdown reaction, it is much better at limiting the reverse reaction than *E. italicus* 2SL; the ratio of catalytic efficiencies of synthesis to breakdown reactions was >10-fold higher for *E. italicus* 2SL (Table 1).

### 2SL enzymes are promiscuous toward small thiols

**Figure 5:**
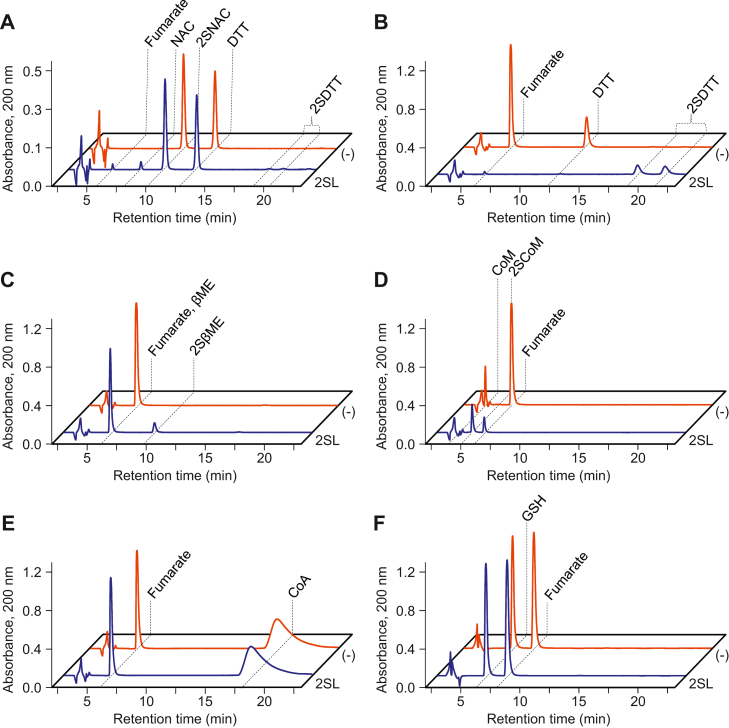
**2SL acts on a variety of small thiol-containing compounds.** Assays (100 μl total volume) contained 50 mM Tris-HCl, pH 7.5, and either 4 mM 2SNAC and 4 mM DTT (*A*) or 4 mM fumarate and either 4 mM DTT (*B*), 4 mM 2-mercaptoethanol (βME) (*C*), 4 mM 2-mercaptoethanesulfonate (CoM) (*D*), 4 mM coenzyme A (CoA) (*E*), or 4 mM GSH (*F*). Reactions were started by adding 1 μg *E.italicus* 2SL (*blue trace*) or an equivalent volume of enzyme storage buffer as a control (*red trace*). After 45 min incubation at 37 °C, reactions were stopped by adding 5 μl of 1 M HCl and analyzed by HPLC. 2SβME, 2SCoM, 2SDTT, 2SNAC, βME, CoA, CoM, DTT, fumarate, GSH, and NAC retention peaks are marked by *dashed lines*. *D. dadantii* 2SL gave nearly identical results. 2SC, *S*-(2-succino)cysteine; 2SL, S-(2-succino) lyase; 2SNAC, *N*-acetyl-2SC; DTT, dithiothreitol; GSH, glutathione; NAC, *N*-acetylcysteine.

While initially optimizing 2SL assays, we included the reducing agent dithiothreitol (DTT) to determine whether it impacted the reaction. When 2SNAC and DTT were incubated with 2SL enzyme, both reaction products NAC and fumarate were detected by HPLC. However, the amount of NAC detected was stoichiometric to 2SNAC loss, while the amount of fumarate detected was 3.2-fold less than expected (Fig. 5*A*). Additionally, DTT levels slightly decreased, and two other peaks appeared that we later determined to be DTT with either one or two *S*-(2-succino) groups covalently attached (Fig. 5*A*). This indicated that 2SL was acting on DTT. To confirm this and to test for other potential substrates, we analyzed synthesis reactions in which fumarate and several low molecular weight thiols were incubated without and with 2SL enzyme. 2SL catalyzed the succination of DTT, 2-mercaptoethanol, and 2-mercaptoethylsulfonate (*i.e.*, coenzyme M, occurring naturally in many microbes) (Fig. 5, *B*–*D*). We could not detect activity against the slightly larger compounds coenzyme A or GSH (Fig. 5, *E* and *F*). Although 2SL enzymes act on several low molecular weight thiols, NAC (or correspondingly 2SNAC) was the best substrate that we tested, as either the amount of enzyme or reaction time had to be increased to reach equilibrium when assaying other substrates.

## Discussion

Our results provide strong biochemical and genetic evidence that *2SL* genes encode a *S*-(2-succino) lyase that is involved in 2SC breakdown. The *2SL* gene replaces *yxeK* in some *yxe* loci (Fig. 1*C*), and our functional complementation assays show that in the context of 2SC breakdown, *yxeK* and *2SL* are functionally redundant (Fig. 2). Both 2SL (Table 1) and YxeK (31) strongly prefer 2SNAC over 2SC as a substrate. Thus, a pathway of 2SC breakdown consisting of acetylation, breakdown, and deacetylation is conserved. YxeK and 2SL represent two variants of the pathway that use completely different enzymatic activities to perform the breakdown step. This general strategy—acetylation, breakdown, and deacetylation—is used for the breakdown of other *S*-alkyl adducts of cysteine such as *S*-methylcysteine (41, 42).

Acetylation of 2SC is a critical step to initiate the breakdown pathway, but it is unclear why acetylation is necessary. Growth of *B. subtilis ΔyxeL* cells, which lack the gene required to acetylate 2SC, was inhibited by exogenous 2SC but not 2SNAC (30). This led to the hypothesis that 2SC is a toxic metabolite and acetylation is required to detoxify the molecule before it is further catabolized. However, the reversibility of 2SL enzymes and their high efficiency at catalyzing the synthesis reaction indicate that acetylation may also be necessary to prevent disruption of intermediary metabolite pools. If 2SL enzymes did not display a strong preference for acetylated substrates, then their expression would deplete endogenous fumarate and cysteine by efficiently converting them to 2SC. This would result in a large accumulation of potentially toxic 2SC. By preferentially acting on NAC, which does not significantly accumulate in some organisms, the synthesis reaction and its impact on fumarate and cysteine pools are minimized.

The enzymes of the *yxe* locus working together catalyze the irreversible breakdown of 2SC. Thus, even though 2SL enzymes kinetically favor the synthesis reaction and 2SNAC is >150-fold more abundant than fumarate and NAC at equilibrium, inclusion in the *yxe* locus suggests that catalyzing the breakdown reaction is the physiological role of *2SL* genes. Remarkably, the *D. dadantii* enzyme has ∼14-fold lower affinity for fumarate than the *E. italicus* enzyme, which may represent an evolved mechanism to limit the occurrence of the back reaction. Other members of the lyase class I conserved protein domain family are also involved in biosynthetic pathways against their thermodynamically favored direction. Adenylosuccinate lyase (E.C. 4.3.2.2) catalyzes a reaction necessary for purine biosynthesis even though at equilibrium (*K*_*c*_ = 0.0068) the substrate strongly outnumbers products (43). Similarly, arginosuccinate lyase (E.C. 4.3.2.1) catalyzes a reaction essential for arginine synthesis in which the equilibrium (*K*_*c*_ = 0.011) strongly favors substrates over products (44). There may be a practical reason for the very low equilibrium constants (*K*_*c*_) of lyase class I family enzymes—it ensures that breakdown (*i.e.*, fumarate release) will not occur unless fumarate levels are low. Thus, the low *K*_*c*_ values of 2SL enzymes prevents a futile cycle by ensuring that 2SC breakdown will not result in overaccumulation of fumarate causing further succination.

It is not known if *yxe* locus-encoded enzymes breakdown endogenously formed 2SC or serve as a means to salvage 2SC from the environment. 2SC has been detected in rat urine (4), so at least one means of introducing 2SC to the environment is known. However, the spontaneous succination of protein cysteine residues is expected to occur in any organism in which fumarate accumulates. Since 2SL enzymes act on 2SNAC and other small molecule thiols but not larger molecules such as GSH and coenzyme A, it is unlikely that 2SL can directly repair succinated proteins. Instead, proteolytic cleavage of protein to release free 2SC would be necessary to salvage the adduct. Regardless of the source of 2SC, identification of two pathway variants for its breakdown underscores the importance of 2SC metabolism.

It is possible that the two pathway variants, with either YxeK or 2SL catalyzing the breakdown step, evolved to operate under different environmental conditions that prefer one reaction over the other. The monooxygenase YxeK requires oxygen to breakdown 2SNAC and might be ineffective under anaerobic conditions (30, 31). 2SL activity should not be affected by oxygen levels directly, but the reverse reaction could become deleterious in response to accumulation of the respiratory intermediate fumarate. Orthologs of YxeK are present in a limited number of bacteria and fungi (30). The distribution of 2SL orthologs is unclear because lyase class I conserved protein domain family enzymes are widely distributed among prokaryotes and eukaryotes and exhibit numerous molecular activities despite having high sequence identity (35, 36). Additional enzymes may also be involved in the breakdown of 2SC, possibly by directly removing the *S*-(2-succino) moiety from damaged proteins. Further investigation is needed to determine the occurrence of 2SC breakdown pathways across life.

Although NAC is strongly preferred as the synthesis reaction substrate, 2SL enzymes are fairly promiscuous and will succinate other small molecule thiols. Additionally, the equilibrium of the reaction strongly favors the production of succinated compounds. These properties make 2SL enzymes ideal catalysts for the synthesis of *S*-(2-succino) compounds. Using 2SL enzymes, we developed simple and highly efficient protocols for synthesizing 2SC, 2SNAC, and other succinated compounds (See Experimental procedures). In principal, 2SL enzymes can also be used to reversibly block thiol groups to prevent their oxidation or participation in thiol Michael addition reactions. 2SL enzymes are good candidates for directed evolution experiments (45) because of their inherent promiscuity and because simple high-throughput colorimetric assays can monitor the succination reaction (46, 47). Thus, a 2SL enzyme could be evolved to succinate a specific thiol-containing chemical of interest. Additionally, 2SL enzymes could be evolved to directly remove the *S*-(2-succino) moiety from damaged proteins.

Metabolite damage of macromolecules is increasingly seen as an important driver of aging (27, 28, 48) and the progression of neurodegenerative diseases such as Parkinson’s (49, 50). In particular, succination of proteins is a major contributor to the pathogenesis of diabetes and certain cancers (51, 52). The identification of a second pathway for 2SC breakdown shows that multiple systems have evolved to deal with this damaged metabolite. Identifying and characterizing systems that deal with 2SC and other macromolecule adducts resulting from metabolite damage will provide important medical insights into the biology of aging and metabolic disease.

## Experimental procedures

### Bioinformatics

Sequences were taken from GenBank or the SEED database (32). Comparative analysis of 2000 representative genomes was performed with SEED and its tools. The full results of the analysis are available in the SEED subsystem named “S-succinylcysteine breakdown.”

### Chemicals

Initially, 2SC and 2SNAC were synthesized and purified as previously described (30). After the discovery of 2SL, succinated compounds were produced enzymatically as follows. 2SNAC was synthesized by combining 1 mmol of sodium fumarate with 1 mmol *N*-Acetyl-L-cysteine in 10 ml of water. After adjusting the pH to 7.5 with NaOH, 10 μg of *D. dadantii* or *E. italicus* 2SL was added, and the reaction was incubated at 37 °C for ∼30 min. Enzyme was removed using an Amicon Ultra-4 10k MWCO spin column (Millipore), and the pH was adjusted to 3.0 with 5M HCl. 2SNAC was purified by HPLC (Agilent 1100 series) using a Hypersil GOLD 250 X 4.6-mm C18 column (ThermoFisher Scientific) with 0.1% TFA and 3% acetonitrile as the mobile phase. Fractions containing 2SNAC (detected by absorbance at 200 nm) were collected, and successive runs were combined, lyophilized, and stored at −20 °C. 2SC and other succinated compounds were synthesized and purified using the same method except that *N*-Acetyl-L-cysteine was replaced with 1 mmol L-cysteine (or other thiol), and the reactions were incubated at 37 °C for 2 to 3 h. All other chemicals were purchased from Sigma-Aldrich. Standards for all substrates and products used in 2SL assays were analyzed by HPLC as previously described to confirm purity and retention times (Fig. S3).

### Constructs for protein expression and complementation assays

The full-length *2SL* coding sequences from *D. dadantii* DSM 18020 (UniProt ID: E0SKP1) and *E. italicus* DSM 15952 (UniProt ID: E6LDH5) were amplified from genomic DNA with Phusion polymerase (New England Biolabs) using primers listed in Table S1. Amplicons were digested with the restriction enzymes NdeI and XhoI (*Dickeya*) or NdeI and BamHI (*Enterococcus*) and ligated into the matching sites of pET28b to facilitate expression of recombinant enzymes containing an *N*-terminal hexahistidine-tag. For complementation assays, coding sequences were amplified as described, and amplicons were digested with SpeI and SmaI (*Dickeya*) or SpeI and BamHI (*Enterococcus*) and ligated into the matching sites of pHCMC04. Additionally, the full-length *yxeK* coding sequence from *B. subtilis* strain 168 was amplified, digested with SpeI and BamHI, and ligated into the matching sites of pHCMC04. All constructs were verified by Sanger sequencing.

### Production and purification of proteins

*E. coli* strain BL21-(DE3)-RIPL harboring each respective expression construct (or empty pET28b vector as a control) was grown at 37 °C in LB medium containing 50 μg/ml kanamycin. When the absorbance at 600 nm reached 0.8, cultures were cooled to 20 °C, and isopropyl β-d-thiogalactoside and ethanol were added to final concentrations of 0.5 mM and 4% (v/v), respectively. Incubation was then continued overnight at 22 °C. Cell lysates were prepared by harvesting cells by centrifugation (8000*g*, 10 min) and suspending pellets in 7 ml of lysis buffer (50 mM Tris-HCl, pH 8.0, 300 mM NaCl, 10 mM imidazole). Cells were sonicated using a Braun-Sonic 2000 set to 50% power for eight, 15 s pulses, cooling on ice for 60 s between pulses. The resulting lysate was centrifuged at 20,000*g* for 10 min. The supernatant was added to a column containing 0.30 ml of HisPur Ni-NTA resin (Thermo Fisher Scientific) and washed with 25 to 30 ml of wash buffer (50 mM Tris-HCl, pH 8.0, 300 mM NaCl, 20 mM imidazole). Recombinant proteins were eluted with 2 ml of elution buffer (50 mM Tris-HCl, pH 8.0, 300 mM NaCl, 200 mM imidazole). Amicon Ultra-4 10k MWCO spin columns were used to concentrate proteins and exchange buffer with 100 mM KCl, 50 mM Tris-HCl, pH 8.0. Glycerol was added to 10% (v/v), and 10 to 20 μl aliquots were snap-frozen in liquid nitrogen and stored at −80 °C. The standard yield of each protein was ∼5 mg per 200 ml culture.

### Enzyme assays

#### HPLC-based assays run to completion

To measure activity in the forward direction (2SNAC→NAC + fumarate; 2SC→Cys + fumarate), assays (100 μl total volume) contained 50 mM Tris-HCl, pH 7.5, 4.0 mM 2SNAC, or 2SC and were started by adding 1.0 μg of either *D. dadantii* or *E. italicus* 2SL enzyme. Assays were incubated at 37 °C for 15 min (2SNAC) or 60 min (2SC) and stopped by adding 5 μl of 1 M HCl. Ten microliter of stopped reaction was analyzed with an Agilent 1100 series HPLC using a Hypersil GOLD 250 × 4.6 mm C18 column (ThermoFisher Scientific) with either 0.1% (w/v) TFA and 3% (v/v) acetonitrile (2SNAC) or 0.1% (w/v) formic acid (2SC) as the mobile phase. Compounds were detected by absorbance at 200 nm. To measure activity in the reverse direction (NAC + fumarate→2SNAC; Cys + fumarate→2SC), assays were performed in the same manner except that 2SNAC or 2SC was replaced with 4.0 mM fumarate and either 4.0 mM NAC or 4.0 mM Cys.

To measure activity against other small molecule thiols, assays were set up as previously described except that 4.0 mM small molecule thiol (DTT, 2-mercaptoethanol, coenzyme A, coenzyme M, or GSH) was combined with 4.0 mM fumarate. Assays were incubated at 37 °C for 45 min and stopped by adding 5 μl of 1 M HCl. Reactions were analyzed by HPLC in the same manner as before using either 0.1% (w/v) TFA and 3% (v/v) acetonitrile (DTT, 2-mercaptoethanol, coenzyme A, or coenzyme M) or 0.1% formic acid (GSH) as the mobile phase. As a control, assays were set up with 2SNAC as before except the reactions were started by adding either 1.0 μg total protein from *E. coli* lysate containing empty pET28b or an equivalent volume of enzyme storage buffer.

#### HPLC-based assays for enzyme kinetics

Assays (100 μl total volume) contained 50 mM Tris-HCl, pH 8.0, appropriate substrates and were started by adding either *D. dadantii* or *E. italicus* 2SL enzyme. A freshly thawed enzyme aliquot was used for all assays used to determine kinetic parameters because we measured a 1% loss of activity every 7 min when our enzyme preparations were stored at 4 °C; enzyme preparations were stable at −80 °C for at least 2 months. Assays were incubated at 21 °C for 2 min before being stopped by adding 5 μl of 1 M HCl. We verified that the levels of substrates and products in stopped reactions remained unchanged for several hours at 4 °C. To determine kinetic parameters for fumarate, assays contained 0.1 μg of enzyme, 4.0 mM NAC, and 0.05 to 8.0 mM fumarate. To determine kinetic parameters for NAC, assays contained 0.1 μg of enzyme, 30 mM fumarate, and 0.03 to 3.0 mM NAC. To determine kinetic parameters for Cys, assays contained 2.0 μg of enzyme, 30 mM fumarate, and 1.0 to 30.0 mM Cys. We verified under these assay conditions that enzyme activity was linear with respect to time and enzyme concentration and that the total amount of substrate(s) consumed was ≤30%. Stopped reactions were analyzed by HPLC as described above, and compounds were detected by absorbance at 200 nm. The amount of compound in each peak was determined by integrating peak areas using OpenLab software (Agilent) and comparing values to those from standard curves prepared for 2SNAC, 2SC, fumarate, NAC, and Cys. Kinetic parameters were calculated by fitting data to the Michaelis–Menten equation using GraphPad Prism Software.

#### Spectrophotometric enzyme assays

Assays (100 μl total volume) contained 100 mM Tris-HCl, pH 8.0, 40 mM glutamate, 2 mM NAD^+^, 6 U malate dehydrogenase from pig heart, 2.5 U aspartate transaminase from pig heart, 2.0 U fumarase from pig heart, and 1 to 40 μg of freshly thawed *D. dadantii* or *E. italicus* 2SL enzyme. Assays were started by adding 0.3 to 5.0 mM 2SNAC or 1.0 to 30.0 mM 2SC, and absorbance at 340 nm was measured with a Cary-UV 3500 spectrophotometer for 2 to 5 min at 21 °C. We verified under these assay conditions that NAD+ levels were stable without addition of substrate, that enzyme activity was linear with respect to time and enzyme concentration, and that the total amount of substrate consumed was ≤10%. Kinetic parameters were calculated by fitting data to the Michaelis–Menten equation using GraphPad Prism Software.

### Complementation analysis

The pHCMC04 plasmids harboring either the *D. dadantii 2SL*, *E. italicus 2SL*, or *B. subtilis yxeK* coding sequences were transformed into a *ΔyxeK B. subtilis* strain (30, 53). The empty vector was also transformed into wildtype *B. subtilis* 168 as a control. Positive transformants were grown overnight in LB medium containing 5 μg mL^−1^ chloramphenicol, washed twice with ED medium without sulfur (8 mM K2HPO4, 4.4 mM KH2PO4, 30 mM NH4Cl, 2.6 mM MgCl2, 27 mM glucose, 0.3 mM Na3-citrate, 0.25 mM L-tryptophan, 0.1 mM FeCl3, 50 μM CaCl2, 5 μM MnCl2, 12 μM ZnCl2, 2.5 μM CuCl2, 2.5 μM CoCl2, and 2.5 μM Na2MoO4) and diluted to absorbances at 600 nm of 1.0, 0.2, 0.04, and 0.008. Five microliter of each dilution was spotted on ED minimal medium without sulfur plates containing 0.8% (w/v) low-melt agarose, 0.2% (w/v) xylose, and either no added sulfur, 0.25 mM 2SC, or 2 mM NaSO4. Plates were incubated at 37 °C for 16 h.

## Data availability

All raw data, bacterial strains, and plasmids are available upon request by contacting Thomas Niehaus: Department of Plant and Microbial Biology, University of Minnesota, Twin Cities, 778 Biological Sciences Bldg., 1445 Gortner Ave, Saint Paul, Minnesota 55,108, Telephone: (612) 301 to 2655; E-mail: tniehaus@umn.edu.

## Supporting information

This article contains supporting information.

## Conflict of interest

The authors declare that they have no conflicts of interest with the contents of this article.
