# Supplementary material for: Identification of a S-(2-succino)cysteine breakdown pathway that uses a novel S-(2-succino) lyase
Source: J Biol Chem. 2022 Oct 27;298(12):102639. doi: 10.1016/j.jbc.2022.102639 (PMC9706529; doi:10.1016/j.jbc.2022.102639)
Supplement: Supplemental Figures S1–S3 and Table S1 [file mmc1.pdf]

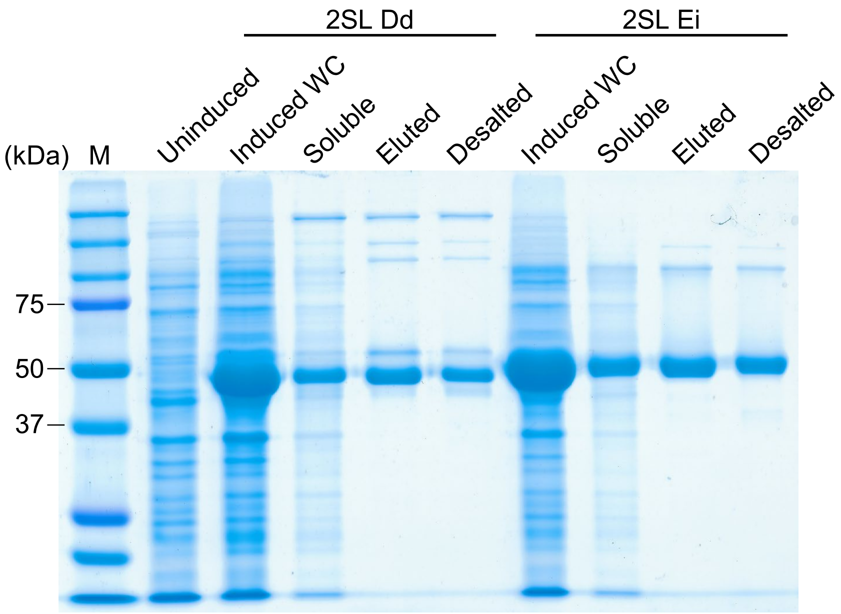

Figure S1: Purification of recombinant 2SL proteins. Recombinant *Dickeya dadantii* (Dd) and *Enterococcus italicus* (Ei) 2SL proteins with *N*-terminal hexahistidine-tags were purified by affinity chromatography and 2 µg of total protein analyzed by SDS-PAGE (12% gels) with Coomassie staining. Whole-cell lysates from uninduced cells and whole-cell and soluble fraction lysates from IPTG-induced cells are shown for comparison. Each desalted preparation was judged to be >90% pure.

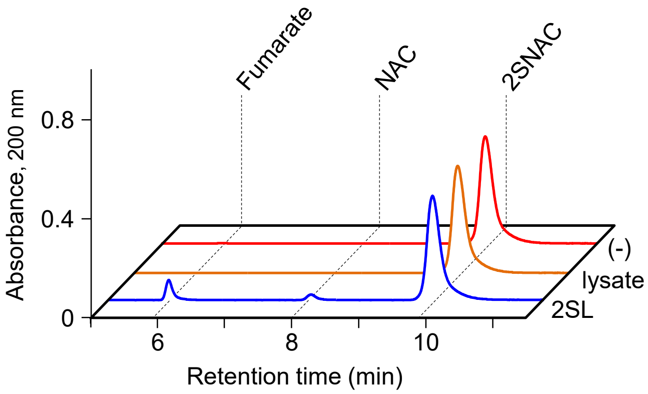

Figure S2: *S*-(2-succino) lyase activity is dependent on recombinant 2SL expression in *E. coli*. Assays (100 µL total) contained 50 mM Tris-HCl, pH 7.5, and 4 mM 2SNAC. Reactions were started by adding 1.0 µg *E. italicus* 2SL (blue trace), 1.0 µg *E. coli* soluble-fraction lysate prepared from IPTG-treated cells containing empty pET28b plasmid (orange trace), or an equivalent volume of enzyme storage buffer (red trace). After incubation for 15 minutes at 37˚C, reactions were stopped by adding 5 µL of 1 M HCl and analyzed by HPLC. 2SNAC, fumarate, and NAC retention times are marked by dashed lines.

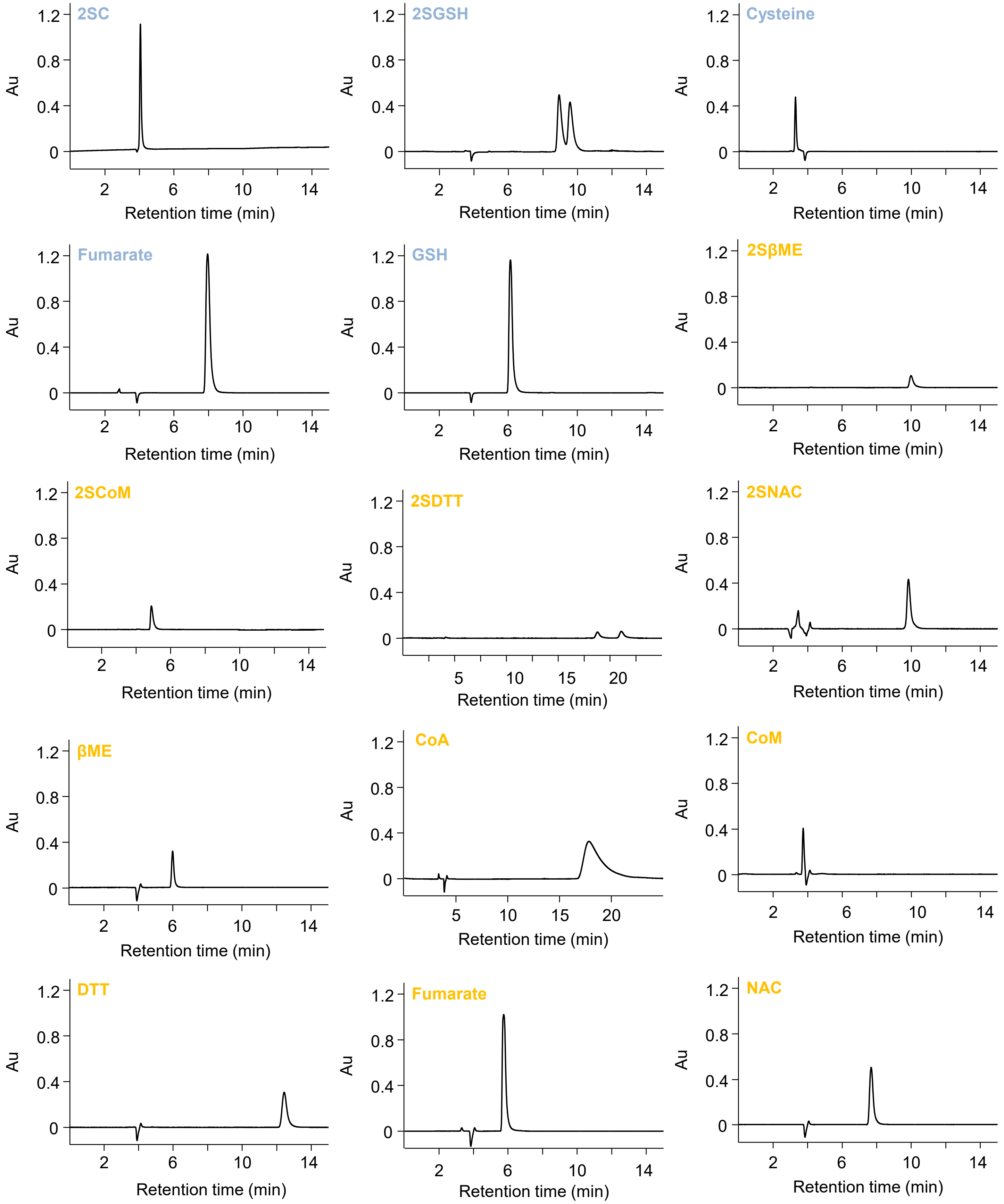

Figure S3: HPLC analysis of substrates and products used in 2SL assays . 40nmol of each compound was analyzed by HPLC, with detection at A_{200}, using either 0.1% formic acid (indicated in blue) or 0.1% TFA and 3% acetonitrile (indicated in yellow) as the mobile phase. 2SC, *S*-(2-succino)cysteine; 2SDTT, *S*-(2-succino)dithiothreitol; 2SβME, *S*-(2-succino)2-Mercaptoethanol; 2SCoM, *S*-(2-succino)2-mercaptoethanesulfonate; 2SNAC, *N*-acetyl-2SC; 2SGSH, *S*-(2-succino)glutathione; CoA, Coenzyme A; CoM, 2-mercaptoethanesulfonate; DTT, dithiothreitol; GSH, glutathione; βME, 2-Mercaptoethanol; NAC, *N*-acetylcysteine.

Supplemental Table 1. Oligonucleotide primers used in this study

Primer Name	Sequence
Functional Complementation Assays	
2SL Dd SpeI F	ggactagtatggcatcacatctgattgattttcttc
2SL Dd SmaI R	tcccccgggttactcggccagcagaccgg
2SL Ei SpeI F	ggactagtatgggatcacacgtaattgatttag
2SL Ei BamHI R	cgcggatccttaaccttccttcaaaaagccttttg
yxeK Bs SpeI F	ggactagtatgacaagcaaaaagaaacaaatc
yxeK Bs BamHI R	cgcggatccttactttgcatagcggtttaccg
Protein expression	
2SL Dd NdeI F	ggaattccatatggcatcacatctgattgattttcttc
2SL Dd XhoI R	ttccgctcgagttactcggccagcagaccgg
2SL Ei NdeI F	ggaattccatatgggatcacacgtaattgatttag
2SL Ei BamHI R	cgcggatccttaaccttccttcaaaaagccttttg
